# Coiled-coil domain containing 109B is a HIF1α-regulated gene critical for progression of human gliomas

**DOI:** 10.1186/s12967-017-1266-9

**Published:** 2017-07-28

**Authors:** Ran Xu, Mingzhi Han, Yangyang Xu, Xin Zhang, Chao Zhang, Di Zhang, Jianxiong Ji, Yuzhen Wei, Shuai Wang, Bin Huang, Anjing Chen, Qing Zhang, Wenjie Li, Tao Sun, Feng Wang, Xingang Li, Jian Wang

**Affiliations:** 1 Department of Neurosurgery, Qilu Hospital of Shandong University and Brain Science Research Institute, Shandong University, #107 Wenhua Xi Road, Jinan, 250012 China; 2 Department of Biomedicine, University of Bergen, Jonas Lies vei 91, 5009 Bergen, Norway; 3 Department of Neurosurgery, Jining No.1 People’s Hospital, Jiankang Road, Jining, 272011 China; 4 Ningxia Key Laboratory of Craniocerebral Diseases, Incubation Base of the National Key Laboratory, Ningxia Medical University, Yinchuan, 750004 Ningxia China

**Keywords:** CCDC109B, HIF1α, Glioma, Proliferation, Invasion

## Abstract

**Background:**

The coiled-coil domain is a structural motif found in proteins that participate in a variety of biological processes. Aberrant expression of such proteins has been shown to be associated with the malignant behavior of human cancers. In this study, we investigated the role of a specific family member, coiled-coil domain containing 109B (CCDC109B), in human gliomas.

**Methods and results:**

We confirmed that CCDC109B was highly expressed in high grade gliomas (HGG; WHO III–IV) using immunofluorescence, western blot analysis, immunohistochemistry (IHC) and open databases. Through Cox regression analysis of The Cancer Genome Atlas (TCGA) database, we found that the expression levels of CCDC109B were inversely correlated with patient overall survival and it could serve as a prognostic marker. Then, a serious of cell functional assays were performed in human glioma cell lines, U87MG and U251, which indicated that silencing of CCDC109B attenuated glioma proliferation and migration/invasion both in vitro and in vivo. Notably, IHC staining in primary glioma samples interestingly revealed localization of elevated CCDC109B expression in necrotic areas which are typically hypoxic. Moreover, small interfering RNA (siRNA) and specific inhibiters of HIF1α led to decreased expression of CCDC109B in vitro and in vivo. Transwell assay further showed that CCDC109B is a critical factor in mediating HIF1α-induced glioma cell migration and invasion.

**Conclusion:**

Our study elucidated a role for CCDC109B as an oncogene and a prognostic marker in human gliomas. CCDC109B may provide a novel therapeutic target for the treatment of human glioma.

**Electronic supplementary material:**

The online version of this article (doi:10.1186/s12967-017-1266-9) contains supplementary material, which is available to authorized users.

## Background

Glioblastoma multiforme (GBM) is the most aggressive malignancy in adults and thus persists as a major unsolved clinical challenge [1]. Despite impressive advances in surgical techniques, radiotherapy and chemotherapy, the median survival time of patients with GBM remains dismally at 14.6 months [2].

Diffuse infiltrative invasion of GBM cells into the adjacent normal brain areas is a major cause of invariable recurrence and relapse after resection of primary tumors [3].

A number of pathological features in GBM provide the basis for understanding the functional consequences of changes in gene expression. For example, hypoxia is a pathological hallmark of GBM. Hypoxia-inducible factor 1 (HIF1), a dimeric transcription factor, is one of the primary regulators that coordinate cellular responses to hypoxia. HIF1 is composed of α and β subunits (HIF1α; HIF1β). HIF1α is rapidly degraded under normoxic conditions but is often stable under hypoxic conditions. However, when HIF1α binds to hypoxia-responsive elements (HREs), it activates transcription of downstream genes, which are involved in tumor angiogenesis, invasion, cell survival, and glucose metabolism [4]. Therefore, identifying HIF1α-targeted molecules will provide further understanding in the development and treatment of human glioma.

Coiled coils are among the most ubiquitous folding motifs identified in proteins and have not only been found in structural proteins but also play a necessary role in various intracellular regulation processes [5]. Coiled coils are involved in signal-transducing events and act as a molecular recognition system. Furthermore, they provide mechanical stability to cells and are involved in movement processes [6]. Increasing evidence suggests that aberrant expression of coiled-coil domain containing proteins influences the migration, invasion and proliferation of various human cancers, including bladder cancer [7], pancreatic cancer [8], gastric cancer [9], papillary thyroid carcinoma [10], leukemia [11], prostate cancer [12], breast cancer [13].

CCDC109B, also known as mitochondrial calcium uniporter b (MCUb), is an MCU isogene [14]. CCDC109B is an evolutionarily conserved protein, which possesses two coiled-coil domains and two transmembrane domains [15]. Functionally, MCUb acts as a negative subunit of the MCU channel, and the MCU/MCUb ratio seems to vary in different tissues, providing a molecular mechanism to mediate the efficiency of mitochondrial calcium (Ca^2+^) intake [16]. The failure of mitochondria to intake calcium leads to the abnormal activation of cytosolic Ca^2+^-dependent enzymes, including calpain proteases [17] and calmodulin-dependent kinases [18] and ultimately leads to changes in cellular signaling cascades which directly regulate cell growth [19], tumor cell invasion [20]. However, the biological significance of CCDC109B in human glioma remains unclear.

Here, we investigated expression of CCDC109B in human glioma tissues and cell lines by analyzing our own cohort and publicly available molecular databases. Then, functional experiments were performed with model systems in vitro and in vivo. We uncovered a potential oncogenic role for CCDC109B in glioma progression and identified HIF1α as a possible transcriptional regulator. These results, support CCDC109B as a new therapeutic target for the treatment of human glioma.

## Methods

### Ethics statement

Human brain tumor (*n* = 68; WHO grade II–IV) and non-neoplastic tissue (*n* = 4) samples were obtained from surgeries performed at the Department of Neurosurgery at Qilu Hospital (Shandong, China). Written informed consent was obtained from all patients, and approval for experiments was obtained from Ethics Committee of the Qilu Hospital. All surgeries and post-operative animal care were approved by the Institutional Animal Care and Use Committee (IACUC) of Shandong University (Shandong, China). Our research complies with the commonly-accepted ‘3Rs’: replacement of animals by alternatives wherever possible, reduction in the number of animals used, and refinement of experimental conditions and procedures to minimize harm to animals.

### Cell culture and hypoxic treatment

Human glioma cell lines, U87MG, U251 and T98 were obtained from the Culture Collection of the Chinese Academy of Sciences (Shanghai, China). The normal human astrocytes (NHA) cell line was a kind gift from the Department of Biomedicine at the University of Bergen (Bergen, Norway). Cells were cultured in Dulbecco’s modified Eagle’s medium (DMEM; Thermo Fisher Scientific, Waltham, MA, USA) supplemented with 10% fetal bovine serum (FBS; Thermo Fisher Scientific) and maintained at 37 °C in a humidified chamber containing 5% CO_2_. For hypoxic treatment, cells were placed in a modulator incubator (HERAcell 150i, Thermo Fisher Scientific) in 94% N_2_, 5% CO_2_, and 1% O_2_. For stable CCDC109B-knockdown, U87MG and U251 cells were infected with lentivirus expressing short hairpin RNA (shRNA) (sh-CCDC109B-1). After 48 h, U87MG or U251 cells were exposed to 0.5 or 2 µg/mL puromycin (A1113802, Thermo Fisher Scientific), respectively, in complete DMEM for an additional 2 weeks. Cells were subsequently treated with PX478 (S7612, Selleck Chemicals; Shanghai, China) and HIF1α siRNA to inhibit HIF1α expression and harvested after 48 h. Sequences of synthesized shRNAs (Genepharma; Shanghai, China) were the following: sh-Negative Control (sh-NC) 5′-TTCTCCGAACGTGTCACGTtt-3′; sh-CCDC109B-1 5′-CAGTCACACCATTATAGTAtt-3′; sh-CCDC109B-2 5′-CTCGACAGGATTATACTTAtt-3′; sh-CCDC109B-3 5′-GCAAGTAGAAGAACTCAATtt-3′. Sequences of synthesized siRNAs (Genepharma) were the following: si-NC 5′-TTCTCCGAAGGTGTCACGG-3′; si-HIF1α-1 5′TACGTTGTGAGTGGTATTATT-3′; si-HIF1α-2 5′-CTGATGACCAGCAACTTGA-3′.

### IHC

Samples were fixed in 4% formalin, paraffin-embedded, and sectioned (4 µm). After de-waxing and rehydration, the sections were incubated with 0.01 M citrate buffer for 20 min at 95 °C for antigen retrieval. Endogenous peroxidase activity and non-specific antigens were blocked with 3% hydrogen peroxide (ZSGB-Bio; Beijing, China) and 10% normal goat serum (ZSGB-Bio) respectively, followed by incubation with primary antibody at 4 °C overnight. Sections were rinsed with phosphate buffered saline (PBS), treated with goat anti-rabbit secondary antibody (ZSGB-Bio), visualized using 3, 3′-diaminobenzidine (DAB, ZSGB-Bio) as substrate, and counterstained with hematoxylin (Beyotime; Haimen, China). Normal mouse serum was used as the negative control. Staining of cancer cells was scored as follows: 0, no staining; 1, weak staining in <50% cells; 2, weak staining in ≥50% cells; 3, strong staining in <50% cells; and 4, strong staining in ≥50% cells. The following primary antibodies (Abcam, Cambridge, UK) were used at the dilutions indicated: CCDC109B (1:200), HIF1α (1:200), Ki-67 (1:500), MMP2 (1:100) and MMP9 (1:200).

### Western blot analysis

Cells and tissues were incubated 30 min in RIPA buffer containing protein inhibitor cocktail for lysis (Thermo Fisher Scientific). After centrifugation and denaturation, protein (20 μg) was separated by 10% polyacrylamide gel electrophoresis and electrophoretically transferred to polyvinylidene difluoride (PVDF) membranes (Merck Millipore; Shanghai, China). Membranes were blocked with Tris Buffered Saline with Tween 20 (TBST, 10 mM Tris, 150 mM NaCl, 0.1% Tween 20) containing 5% bovine serum albumin (BSA, Thermo Fisher Scientific),and incubated overnight at 4 °C with the following primary antibodies against CCDC109B (1:500), HIF1α (1:1000), MMP2 (1:1000), MMP9 (1:1000) and β-Tubulin (1:1000; Cell Signaling Technology; Danvers, MA, USA). Membranes were incubated the next day with secondary antibody (1:5000; Santa Cruz; Dallas, TX, USA) conjugated to horseradish peroxidase (HRP) for 1 h at room temperature. Proteins were quantified using a system for detecting chemiluminescence (Bio-Rad; Irvine, CA, USA), according to the manufacturer’s protocol. Representative images and data were obtained from at least three independent biological replicate experiments.

### Cell migration and invasion assay

Cell migration and invasion assays were performed in uncoated and matrigel-coated (BD Biosciences; San Jose, CA, USA) Transwell chambers (8 μm pores; Corning Costar; Corning, NY, USA). Cells (2 × 10^4^) in medium (200 µL) with 1% FBS were seeded in the top chamber. The lower chamber was filled with medium (600 µL) containing 30% FBS. Chambers were incubated for 24 h under normoxic or hypoxic conditions. Cells that migrated to or invaded into the lower surface were fixed with 4% paraformaldehyde (Solarbio; Beijing, China), stained with crystal violet (Solarbio) for 15 min and counted under bright field microscopy. Images were acquired from 5 random fields in each well, and cell numbers were determined using Kodak MI software. Each experiment was repeated three times in triplicate.

### Immunofluorescence

To assess the distribution and expression levels of CCDC109B, NHA and glioma cells were seeded onto glass slides. The cells were then washed twice with PBS and fixed with 4% paraformaldehyde for 20 min at room temperature. Cells were rinsed with PBS, permeabilized with 0.5% Triton X-100 (Solarbio) for 15 min, and blocked with 10% normal goat serum for 60 min at room temperature. Cells were stained with primary antibody against CCDC109B (1:100) at 4 °C overnight, followed by incubation with Alexa Fluor 594 goat anti-rabbit IgG (Abcam, UK; 1:800) for 1 h at room temperature. Cell nuclei were stained with DAPI (Sigma-Aldrich, Germany) at 37 °C for 10 min, and images were obtained with confocal microscopy (LSM780, Zeiss).

### Proliferation assay

Cell proliferation was measured using the EdU Apollo 567 Cell Tracking Kit (Ribo-bio; Guangzhou, China). Cells (2 × 10^4^) under different treatments were seeded onto 24-well plates, exposed to 200 μM of 5-ethynyl-20-deoxyuridine for 2 h at 37 °C, fixed with 4% paraformaldehyde for 20 min, and treated with 0.5% Triton X-100 for 10 min. Cells were rinsed with PBS three times, and incubated with 100 μL of Apollo reagent for 30 min. Nuclie were stained with Hochest33342. The percentages of EdU-positive cells were determined from 500 cells and three independent experiments were performed.

### Plate colony forming assay

NC and sh-CCDC109B-1 glioma cells were seeded onto six-well plates (120 cells per well) and cultured for 2 weeks in medium that was changed twice each week. Colonies of more than 50 cells were counted after fixation and staining with 100% methanol and 5% crystal violet. Data reported represent the average of three independent experiments.

### Quantitative real-time PCR

Total RNA was isolated from cells using Trizol reagent (Takara; Tokyo, Japan) according to the manufacturer’s protocol. Total RNA was reverse-transcribed, and the resulting cDNA was used as template in real-time quantitative PCR performed with the standard SYBR premix Ex Taq (Takara) on the Real Time PCR Detection System (480II, Roche; Pleasanton, CA, USA). GAPDH served as an internal control, and independent experiments were conducted in triplicate. The following primers were used: GAPDH, forward, 5′-AATGAAGGGGTCATTGATGG-3′, reverse, 5′-AAGGTGAAGGTCGGAGTCAA-3′; HIF1α, forward, 5′-TGGCAGCAACGACACAGAAA-3′, reverse, 5′-TGCAGGGTCAGCACTACTTC-3′; CCDC109B, forward, 5′-ACACTGCTGAGATGGAACACAT-3′, reverse, 5′- TTGGCTTCCGAATGAGCTTCTA-3′.

### Animal studies

For generation of the subcutaneous GBM model, female 4-week-old nude mice (SLAC laboratory animal Center; Shanghai, China) were maintained in a barrier facility on high-efficiency particulate air (HEPA)-filtered racks. Digoxin and saline were purchased from Qilu Hospital, Shandong University. Nude mice (*n* = 16) were divided into two groups (U87MG + saline, U87MG + digoxin, 8 mice per group). Cells were harvested by trypsinization, resuspended at 10^7^ cells/mL in a 1:1 solution of PBS/Matrigel (BD Biosciences, USA), and injected subcutaneously into the right shoulder of the mouse. The tumor tissues were isolated 37 days after injection, and then used for protein extraction.

For orthotopic xenografts, 4-week-old female nude mice (*n* = 16) were divided into two groups (sh-CCDC109B-1 and NC group), and U87MG or U87MG modified cells (1 × 10^6^) were implanted into the brain using a stereotactic apparatus (KDS310, KD Scientific; Holliston, MA, USA). Animals which displayed symptoms such as severe hunchback posture, apathy, decreased motion or activity, dragging legs, or drastic loss of body weight were euthanized by cervical dislocation. Excised tumor tissues were formalin-fixed, paraffin-embedded, and sectioned for Hematoxylin–Eosin (HE) staining and IHC.

### Statistical analysis

All data are presented as a mean ± the standard error of the mean (S.E.M). The Student’s t test was used when only two groups were being compared. Analysis of variance (ANOVA) was used in cases where there were more than two groups being compared. Survival curves were estimated by the Kaplan–Meier method and compared using the log-rank test. For multivariate analysis, independent prognostic factors were determined using the Cox’ proportional hazards model. Variables that might be dependent on other variables were excluded from the model. A two-tailed χ^2^ test was used to determine the association between CCDC109B and HIF1α. GraphPad Prism version 7.00 software program (GraphPad; La Jolla, CA, USA) was used to analyze in vitro and in vivo experiments. Differences were considered to be statistically significant when *P* < 0.05.

## Results

### CCDC109B is highly expressed in high grade gliomas

**Fig. 1:**
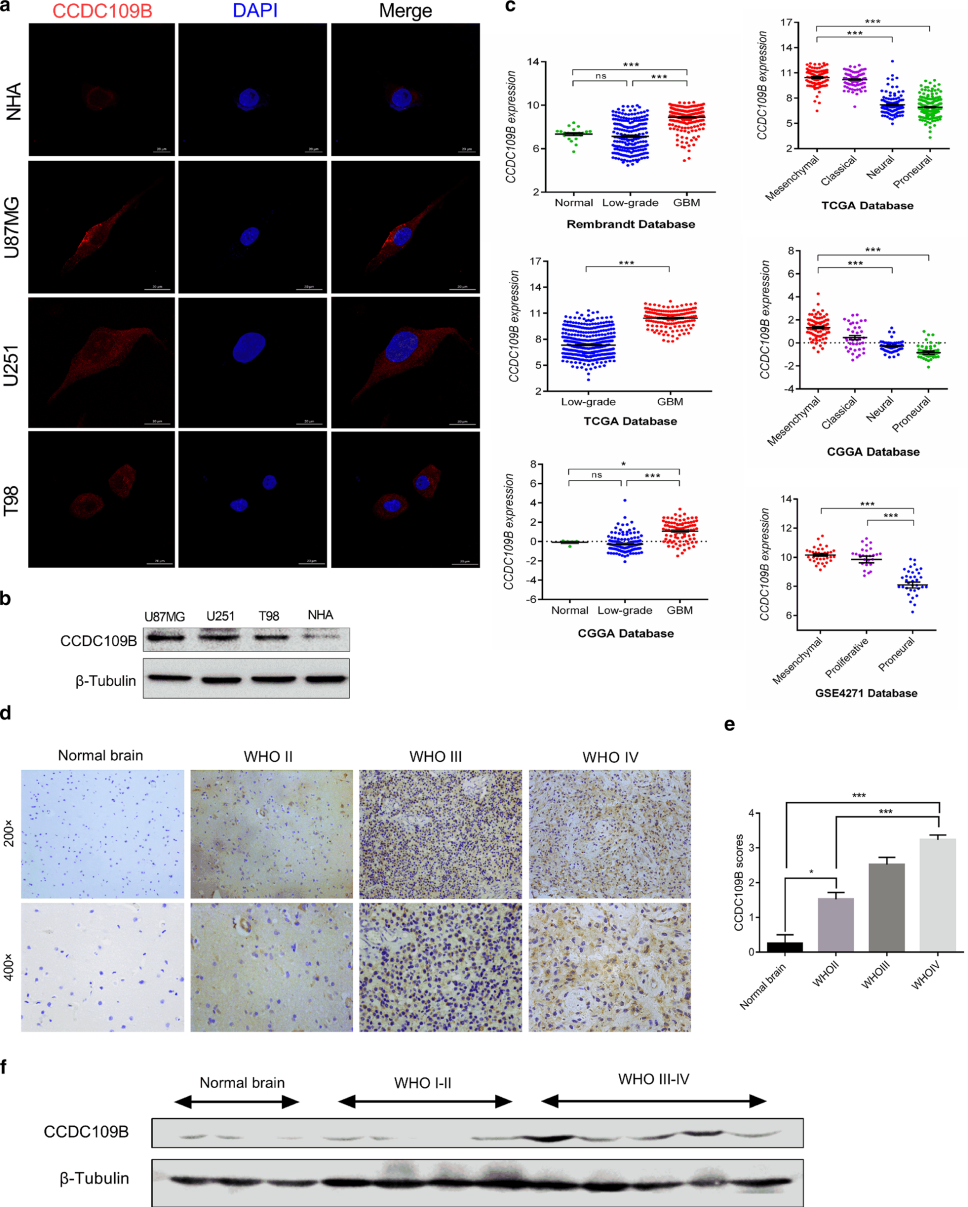
CCDC109B is highly expressed in high grade gliomas. **a** Images of immunofluorescence performed with an antibody against CCDC109B (*red*) in NHA, U87MG, U251, T98 cells and visualized using confocal microscopy. Nuclei were labeled with DAPI (*blue*). *Scale bar* 20 μm. **b** Western blot analysis of CCDC109B levels in U87MG, U251, T98 and NHA. **c** mRNA expression levels of *CCDC109B* as determined using TCGA, CGGA, GSE4271 and Rembrandt databases. **d** Representative images of IHC staining with anti-CCDC109B antibody on human glioma and non-neoplastic brain tissue samples. *Magnification* ×200, upper; ×400, lower. **e** Graphical representation of scoring performed on IHC staining of glioma and non-neoplastic tissue samples for CCDC109B levels. *Bar graphs* show the mean ± the standard error of the mean (SEM) for each group. **f** Western blot analysis of CCDC109B in lysates (20 µg) prepared from different grades of human gliomas (WHO grades II–IV) and normal brain tissues (*ns* not significant, *P < 0.05, ***P < 0.001)

Immunofluorescence staining were used to detect localization and expression level of CCDC109B in NHA cell line and human glioma cell lines in vitro. The results revealed cytoplasmic localization and increased expression levels of CCDC109B protein in U87MG, U251 and T98 glioma cells compared to NHA (Fig. 1a). Western blot analysis confirmed the cell staining. Expression levels of CCDC109B protein was increased in glioma cell lines relative to NHA in vitro (Fig. 1b). To further confirm the level of CCDC109B in normal brain tissue samples and different grades glioma tissues, we searched publicly available databases, Rembrandt, TCGA, Chinese Glioma Genome Atlas (CGGA) and found a relatively higher mRNA level of *CCDC109B* in HGG in contrast to low grade gliomas (LGG; WHOI-II) and normal brain tissues (*P* < 0.001, Fig. 1c). Expression levels of CCDC109B were also stratified on the basis of the molecular subtypes of human glioma (mesenchymal, classical, neural, and proneural) in TCGA, CGGA and Gene Expression Omnibus (GSE4271) databases. Intriguingly, CCDC109B was increased in the mesenchymal glioma molecular subtype compared to other subtypes (*P* < 0.001, Fig. 1c), which indicates a potential role of CCDC109B expression in glioma migration and invasion. We validated the results of our molecular analysis in a cohort of glioma and non-neoplastic brain tissue samples from our own institution using IHC and western blot analysis. CCDC109B protein was highly expressed (scores ≥ 3) in majority of HGG (29/49, 59.2%) and very few LGG (2/19, 10.5%), with almost no expression in normal brain tissue samples (*n* = 4; Fig. 1d, e). The difference in expression levels between these groups was statistically significant (*P* < 0.001, Table 1), with high CCDC109B expression correlating with increased tumor grade (*P* < 0.001, Fig. 1e). Expression by western blot corroborated these results. CCDC109B protein levels were increased in HGG cases (*n* = 5) relative to normal brain tissues (*n* = 3) and LGG (*n* = 4; Fig. 1f). These results all together indicated that CCDC109B levels were elevated in HGG compared to LGG and non-neoplastic brain tissue samples.

**Table 1:** Relationship between *CCDC109B* expression levels and clinicopathological features in glioma

Variables	No. of cases	*CCDC109B* expression		*P* value
		Low	High	
Age (year)				
<60	40	21	19	0.9306
≥60	28	15	13	
Gender				
Male	32	17	15	0.4747
Female	36	16	20	
Tumor size (cm)				
<4	35	19	16	0.1381
≥4	33	12	21	
Cystic change				
Absent	29	15	14	0.7012
Present	39	22	17	
Edema				
None to mild	45	23	22	0.7977
Moderate to severe	23	11	12	
WHO grade				
II	19	17	2	
III	23	20	29	0.0003
IV	26			

### CCDC109B is a prognostic marker in glioma patients

**Fig. 2:**
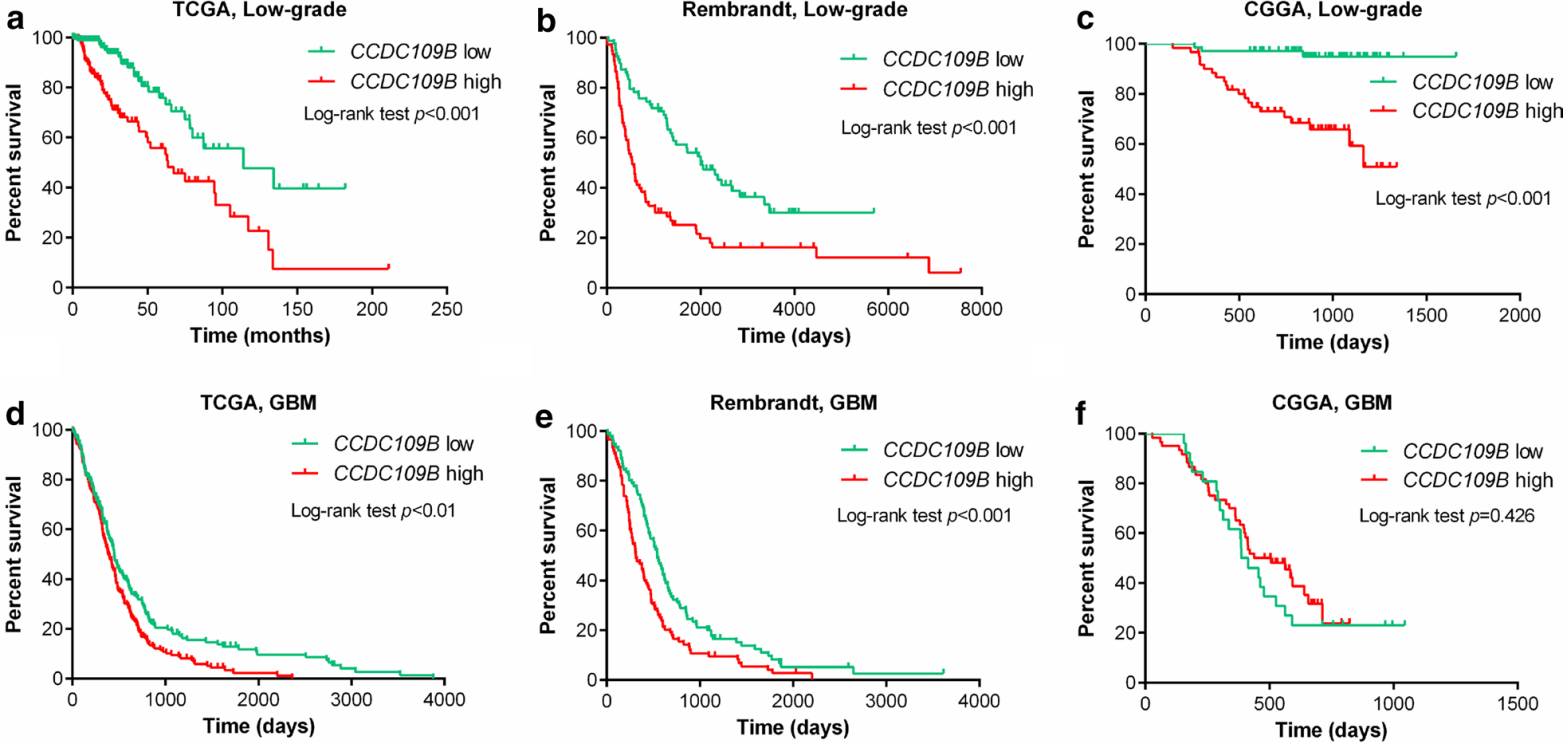
CCDC109B is a prognostic marker in glioma patients. **a**–**c** OS analysis of CCDC109B^low^ and CCDC109B^high^ groups in LGG patients from TCGA, Rembrandt and CGGA databases. **d**–**f** OS analysis of CCDC109B^low^ and CCDC109B^high^ groups in GBM from TCGA, Rembrandt and CGGA databases

The difference in expression levels of CCDC109B between HGG and LGG drove us to further investigate whether CCDC109B could serve as a prognostic marker in glioma patients. We analyzed the relationship between CCDC109B level and overall survival (OS) of glioma patients in TCGA, Rembrandt and CGGA databases based on tumor grade. LGG patients with a high or low expression of CCDC109B displayed a considerably different median OS in all three databases (all *P* < 0.001, Figs. 2a–c). Furthermore, levels of CCDC109B also exhibited a significant inverse relationship with median survival time of GBM patients in TCGA (*P* < 0.01, Fig. 2d) and Rembrandt (*P* < 0.001, Fig. 2e) databases. This correlation however was not significant in GBM patients from the CGGA database (*P* = 0.426, Fig. 2f).

To further confirm the prognostic value of CCDC109B in glioma, univariate Cox analysis was performed with clinical and molecular data of glioma patients in TCGA. The results demonstrated that age (HR = 1.075, *P* < 0.001), WHO grade (HR = 9.560, *P* < 0.001), *CCDC109B* expression (HR = 1.861, *P* < 0.001), and mutation status of isocitrate dehydrogenase 1 (*IDH1*, HR = 0.244, *P* < 0.001), were all prognostic indicators for glioma patients (Table 2).

**Table 2:** Univariate analysis of variables related to OS in patients from TCGA

Variable	Univariate Cox regression	
	HR (95% CI)	*P* value
Age	1.075 (1.063–1.088)	<0.001
Increasing years		
Gender	0.992 (0.737–1.334)	0.957
Female vs male		
WHO grade	9.590 (6.849–13.427)	<0.001
GBM vs low-grade		
CDCC109B expression	1.861 (1.699–2.038)	<0.001
High vs low		
*IDH1* status	0.095 (0.067–0134)	<0.001
Mutation vs wild-type		

*HR* hazards ratio, *CI* confidence interval

### Knockdown of CCDC109B inhibits proliferation, migration, and invasion of glioma cells in vitro

**Fig. 3:**
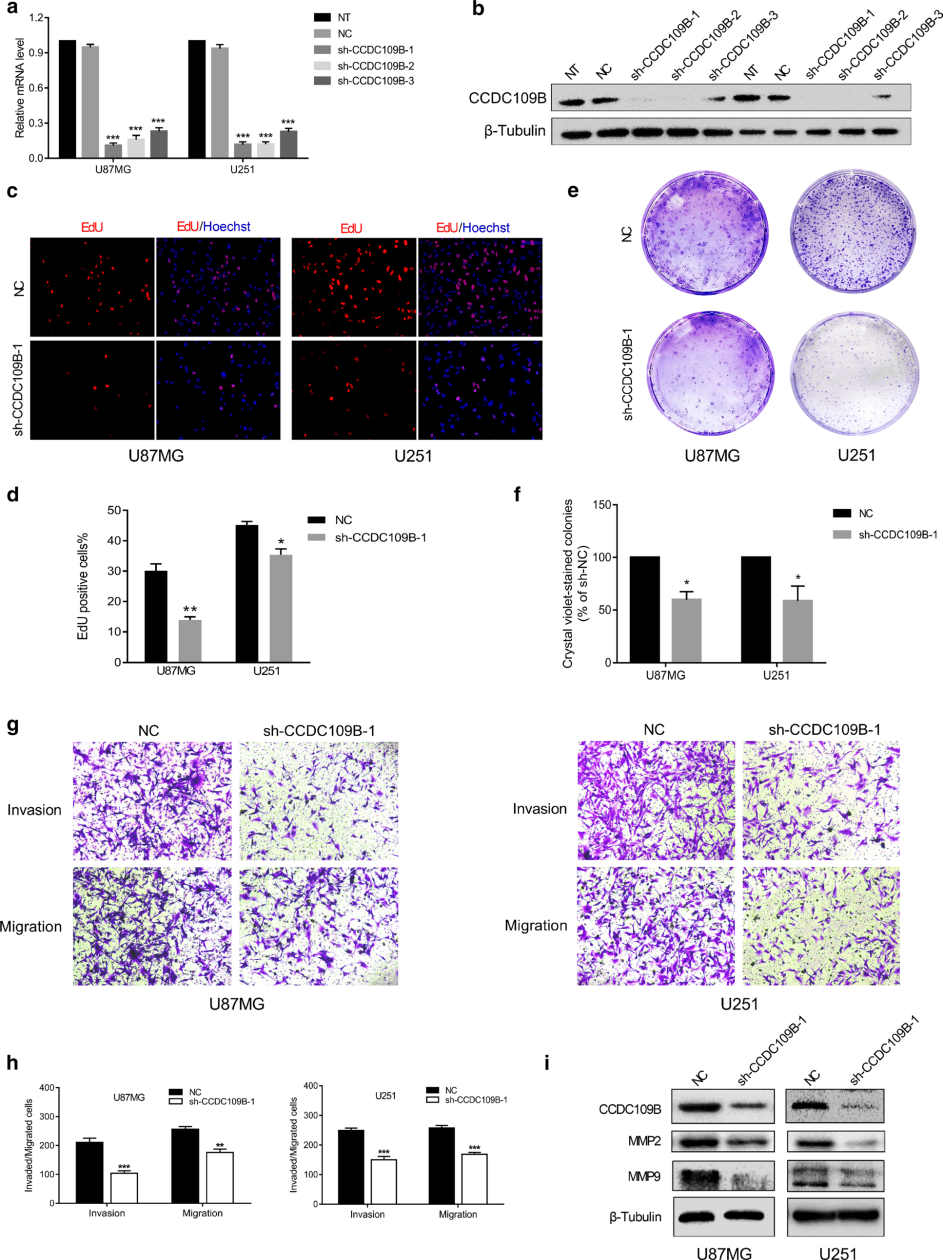
Knockdown of CCDC109B inhibits proliferation, migration, and invasion of glioma cells in vitro. Knockdown efficiency of CCDC109B in U87MG and U251 cells was determined in **a** by qRT-PCR and in **b** by western blot analysis; **c** EdU assays for U87MG- and U251-NC or sh-CCDC109B-1 cells. *Magnification* ×200. **d** Graphic representation of ratios of EdU positive cells in U87MG- and U251-NC and sh-CCDC109B-1 cells. Data are presented as the mean ± SEM. **e** Representative images of colony forming assays for U87MG- and U251-NC (*top*) or shCCDC109B-1 cells (*bottom*). **f** Graphic representation of colony forming results in U87MG- and U251-NC and -sh-CCDC109B-1. Data are presented as the mean ± SEM. **g** Images of Transwell migration and invasion assays performed with U87MG- and U251-NC and sh-CCDC109B-1 expressing cells. *Magnification* ×100. **h** Graphic representation of cell counts from Transwell assays after a 24 h incubation. Experiments were performed in triplicate and counted from 5 random fields. Data are presented as the mean ± SEM. **i** Western blot analysis for the expression of MMP2 and MMP9 in NC and sh-CCDC109B-1 U87MG and U251 glioma cell lines (*P < 0.05, **P < 0.01, ***P < 0.001)

To determine whether the protein has a biological role in glioma, we designed lentiviral constructs expressing shRNAs targeted against CCDC109B for stably knockdown of expression. Compared to NC constructs, the mRNA levels of *CCDC109B* in U87MG and U251 cells were significantly down-regulated after infection with three different shRNAs targeting CCDC109B (sh-CCDC109B-1; sh-CCDC109B-2; sh-CCDC109B-3; *P* < 0.001, Fig. 3a). Protein was nearly undetectable in cells infected with sh-CCDC109B-1 (Fig. 3b). Therefore, this shRNA was used in subsequent functional assays.

We evaluated the effects of CCDC109B knockdown on glioma cell proliferation using EdU (Fig. 3c) and plate colony forming assays (Fig. 3e). Loss of CCDC109B led to significant decreases in the percentage of EdU positive cells (all *P* < 0.05, Fig. 3d) and colony forming ability (all *P* < 0.05, Fig. 3f) in both U87MG and U251 cells.

In Transwell migration and invasion assays (Fig. 3g), CCDC109B knockdown attenuated the number of U87MG and U251 cells that had migrated/invaded after a 24-h incubation (all *P* < 0.05, Fig. 3h). Western blot analysis revealed that MMP2 and MMP9, two metalloproteinases which play critical roles in tumor invasion and migration [21, 22], were also reduced after CCDC109B knockdown (Fig. 3i). Taken together, these functional assays indicated that expression levels of CCDC109B potentially promoted glioma cell proliferation, migration and invasion in vitro.

### Knockdown of CCDC109B suppresses glioma progression in vivo

**Fig. 4:**
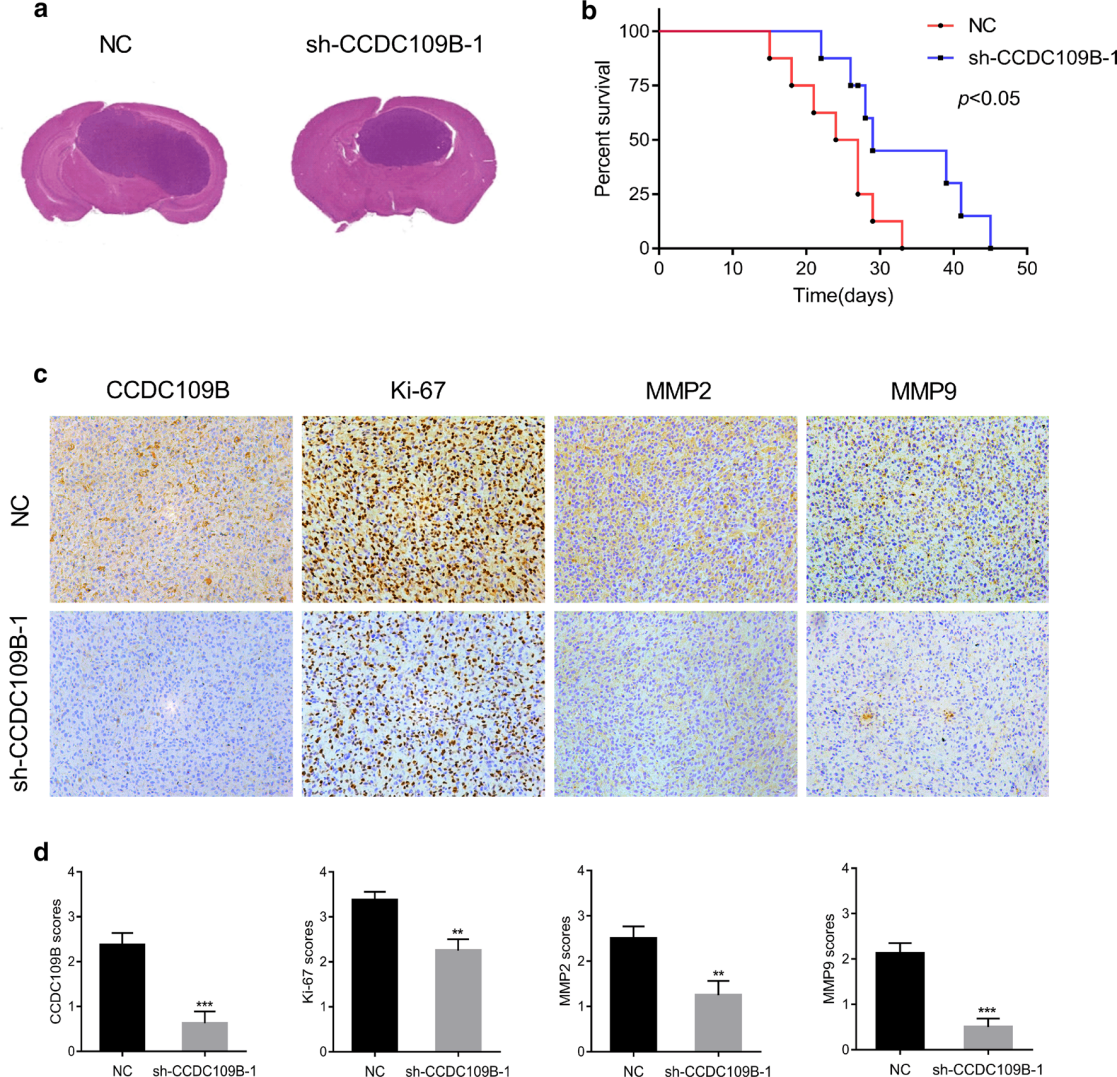
Knockdown of CCDC109B suppresses glioma progression in vivo. **a** HE staining of orthotopic xenografts to verify brain tumor volume. **b** Kaplan–Meier survival analysis performed with survival data of mice implanted with U87MG-NC and -sh-CCDC109B-1 cells. Log-rank test was used to calculate *P* values which were <0.05. **c** Representative IHC images of CCDC109B, Ki-67, MMP2 and MMP9 expression in xenografts sections of cells indicated. *Magnification* ×200. **d** Graphic representation of IHC scoring of CCDC109B, Ki-67, MMP2, MMP9 expression in xenograft sections generated from NC and sh-CCDC109B-1 expressing U87MG cells. Data are presented as the mean ± SEM (**P < 0.01, ***P < 0.001)

We next established orthotopic tumor models by implanting U87MG-NC cells or U87MG-sh-CCDC109B-1 cells intracranially in nude mice to investigate whether CCDC109B mediated proliferation and invasion of glioma cells in vivo. Tumor volume was decreased with CCDC109B knockdown (Fig. 4a) and OS was prolonged in mice when compared to controls (*P* < 0.05, Fig. 4b). IHC staining for CCDC109B, and markers for proliferation (Ki-67), and invasion (MMP2 and MMP9) performed on sections from xenografts further established a potential role for CCDC109B in regulating these pathways (Fig. 4c). Lower levels of all three markers, Ki-67, MMP2, and MMP9, were observed in xenografts of U87MG-sh-CCDC109B-1 compared to controls (all *P* < 0.01, Fig. 4d).

### CCDC109B expression is induced by hypoxia and regulated by HIF1α

**Fig. 5:**
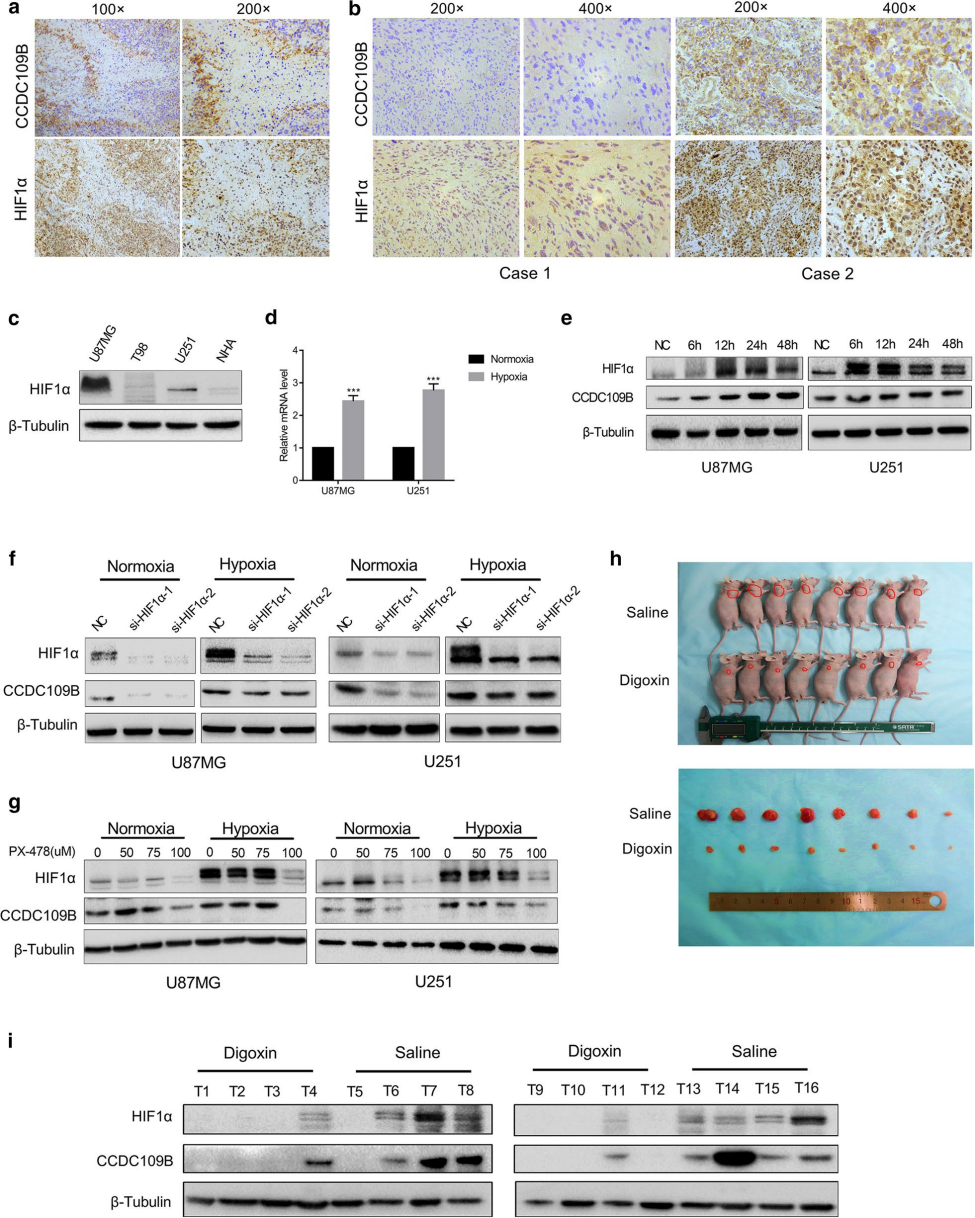
CCDC109B expression is induced by hypoxia and regulated by HIF1α. **a** Representative IHC images of CCDC109B and HIF1α in primary human GBM tissues. *Magnification* ×100 and ×200. **b** Analysis of HIF1α and CCDC109B expression in human GBM tissues by IHC staining. *Magnification* ×200 and ×400. Representative images were labeled as case 1 and case 2. **c** Western blot analysis of HIF1α in U87MG, T98, U251 and NHA. **d** qRT-PCR were used to determine mRNA levels of *CCDC109B* in U87MG or U251 cells cultured under normoxic or hypoxic conditions. **e** Western blot analysis for HIF1α and CCDC109B protein levels in U87MG and U251 cells cultured under hypoxia for the indicated time. **f** Western blot analysis for HIF1α and CCDC109B in U87MG and U251 cells transfected with NC, si-HIF1α-1 or si-HIF1α-2 under normoxic or hypoxic conditions for 48 h. **g** Western blot analysis for HIF1α and CCDC109B in U87MG and U251 cells treated with PX478 (0, 50, 75, 100 μM) and cultured under normoxic or hypoxic conditions for 48 h. **h** Representative images of implanted nude mice injected intraperitoneally with saline or digoxin (2 mg/kg) every day for one month. Images for corresponding subcutaneous U87MG xenografts after surgical removal are also shown. **i** Western blot analysis to determine levels of HIF1α and CCDC109B in tumors from nude mice treated with saline or digoxin. Data are presented as the mean ± SEM (***P < 0.001)

One of the unexpected findings from IHC performed on primary GBM samples was the high expression of CCDC109B localized in areas bordering necrosis. Increased expression of HIF1α, a transcriptional regulator typically induced by hypoxia, was also increased in these areas (Fig. 5a). IHC staining was used to further examine the relationship between HIF1α and CCDC109B in a cohort of GBM specimens (*n* = 32; Fig. 5b; Additional file 1: Table S1; *P* = 0.020).

We next wanted to establish whether HIF1α might induce CCDC109B under hypoxia. We selected glioma cell lines, U87MG and U251, to further examine the relationship between these two proteins, as they express higher levels of HIF1α protein than T98 or NHA (Fig. 5c). We cultured U87MG and U251 cells under hypoxia (1% O_2_) for 6, 12, 24 and 48 h. mRNAs levels of *CCDC109B* were increased by ~twofold under hypoxia (*P* < 0.001, Fig. 5d), and coordinate increases in CCDC109B and HIF1α at the protein level were confirmed by western blot (Fig. 5e). U87MG and U251 cells were treated with siRNAs targeting HIF1α (si-HIF1α and si-HIF1α-2) or an inhibitor of HIF1α (PX478) [23–25] to test whether HIF1α is involved in regulating CCDC109B expression. Down-regulation of HIF1α reduced mRNA levels of *CCDC109B* (Additional file 2: Figure S1A, B) and led to moderate decreases in CCDC109B protein (Fig. 5f, g).

To verify these results in vivo, we implanted U87MG into the right shoulder of nude mice to establish subcutaneously xenografts. Digoxin, a drug widely used to inhibit HIF1α activity [26–28], was subsequently injected into implanted animals to investigate whether HIF1α induced CCDC109B in vivo. Mice were injected one week after implantation with saline or digoxin (2 mg/kg) intraperitoneally every day for 30 days. Tumor size was significantly larger in the saline than the digoxin treated animals (Fig. 5h). We next measured protein levels of HIF1α and CCDC109B in treated and untreated xenografts by western blot. CCDC109B expression was decreased in digoxin relative to saline treated animals (Fig. 5i). Taken together, these results demonstrated that hypoxia enhanced CCDC109B expression and that HIF1α potentially induced expression of CCDC109B.

### CCDC109B knockdown inhibits hypoxia-induced migration and invasion of glioma cells

**Fig. 6:**
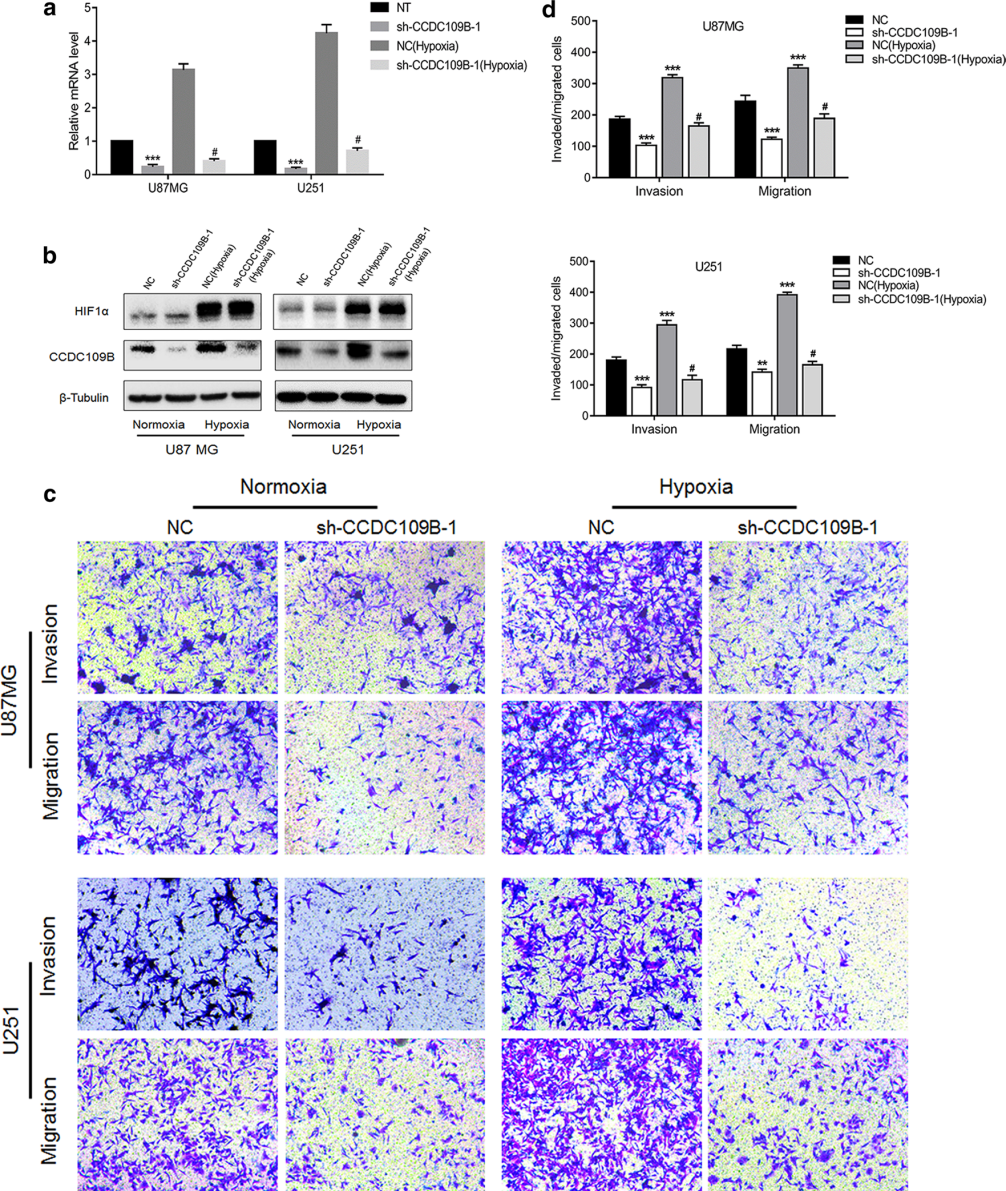
CCDC109B knockdown inhibits hypoxia-induced migration and invasion of glioma cells. U87MG- and U251-NC or -sh-CCDC109B-1 cells were cultured under normoxic or hypoxic conditions for 48 h and the following assays were performed. **a** qRT-PCR to detect expression of *CCDC109B* expression; **b** Western blot analysis for CCDC109B and HIF1α in cell type indicated. **c** Representative images of Transwell migration and invasion assays performed on U87MG- and U251-NC and -sh-CCDC109B-1 cells. Cells were seeded into chambers and incubated under normoxia or hypoxia for 24 h. **d** Graphic representation of cell counts from Transwell assays. Migraded/invaded cells were counted and averaged for each experimental condition. Data are presented as the mean ± SEM (**P < 0.01, ***P < 0.001 vs NC group; ^#^indicates *P* < 0.001 vs NC under hypoxia)

We next investigated whether CCDC109B knockdown altered hypoxia-induced migration and invasion of U87MG and U251 cells. Knockdown of CCDC109B in glioma cells under hypoxia was confirmed by qRT-PCR and western blot analysis (Fig. 6a, b). In Transwell invasion and migration assays, hypoxia significantly enhanced invasion and migration of U87MG and U251 cells (Fig. 6c, d). In contrast, glioma cell migration and invasion was significantly attenuated in U87MG- and U251-sh-CCDC109B-1 cells (all *P* < 0.01, Fig. 6c, d). These results indicated that CCDC109B promoted hypoxia-induced invasion and migration in human glioma cell lines U87MG and U251 in vitro.

## Discussion

Over the past decades, rapid advancement in technologies has enabled us to describe human gliomas with greater molecular detail. However, the value of established biomarkers is limited. In this regard, identification of new molecular targets and a better understanding of underlying pathways might improve the prognosis and the efficiency of treatment for glioma patients. In the present study, we found that CCDC109B was highly expressed in HGG relative to LGG and normal brain tissues. Silencing of CCDC109B inhibited glioma proliferation, migration and invasion of glioma cells in vitro and led to decreased tumor volume and prolonged OS in vivo. Unexpectedly, we found CCDC109B expression to be drastically upregulated under hypoxia and that subsequent knockdown inhibited hypoxia-induced migration and invasion of glioma cells. Finally, functional disruption with siRNAs revealed HIF1α as a potential transcriptional regulator of CCDC109B expression both in vitro and in vivo. Our study for the first time identifies CCDC109B as a potential tumor promotor in glioma progression and provides rational for targeting CCDC109B as novel treatment or prognostic marker in human glioma.

CCDC109B was first identified as a paralogue of MCU, with two predicted transmembrane domains. In Hela cells, CCDC109B acts as a dominant negative mediator of MCU, attenuating mitochondria calcium increases evoked by agonist stimulation [16]. In this study, we found that CCDC109B expression was elevated in HGG tissues and observed high expression level of CCDC109B in human glioma cell lines. Then, analysis of publicly available data revealed that increased expression of *CCDC109B* mRNA level was highly associated with the mesenchymal molecular subtype in human glioma. Next, we confirmed this finding in a cohort of glioma and non-neoplastic brain tissue samples. Consistent with our results, higher expression of CCDC109B in GBM was reported in a meta-analysis performed with a large cohort [29]. In addition, results from gene profiling analysis conducted by another group revealed increased CCDC109B as a possible factor contributing to/associated with temozolomide (TMZ) resistance in malignant gliomas [30]. Finally, CCDC109B overexpression has also been reported in leukemia [31]. All together, these results indicate that CCDC109B might function as an oncogene in human gliomas and possibly other cancers as well.

Importantly, we took our molecular analysis a step further and examined the functional consequences of inactivating CCDC109B with shRNAs in human glioma cell lines. Our data demonstrated that knockdown of CCDC109B significantly attenuated proliferation, migration and invasion of glioma cells in vitro and led to decreased tumor volume and prolonged OS of tumor-bearing mouse in orthotopic models. Moreover, we demonstrated that decreased expression of MMP2 and MMP9, proteins linked to invasion/migration accompanied CCDC109B knockdown. Mounting evidence suggests that a critical role of coiled-coil motif proteins in human tumorigenesis is in their mediation of cellular processes, mainly proliferation and invasion [6, 29, 30]. As one member of the family of coiled-coil motif proteins, CCDC109B plays an important role in facilitating Ca^2+^ flux across the inner mitochondrial membrane (IMM) [14]. Aberrant expression of CCDC109B has been shown to lead to mitochondrial Ca^2+^ remodeling and the subsequent activation of signaling cascades associated with cancer formation and maintenance [32]. Our results parallel a study conducted by Flotho et al. [31] where investigators demonstrated that CCDC109B regulates cell proliferation and predicts treatment outcome in childhood acute lymphoblastic leukemia. Collectively, we and others have demonstrated that CCDC109B contributes to glioma and possibly more generally to cancer development by promoting cellular processes such as proliferation and invasion/migration.

An unexpected finding in our study was that CCDC109B expression was induced by hypoxia. Intratumoral hypoxia, which plays a key role in tumor angiogenesis, growth and invasion, has been directly associated with an aggressive phenotype of GBM [33, 34]. HIF1α, is a critical mediator of cellular response to hypoxia and therefore has been found to be involved in cancer progression and metastasis [35, 36]. Inhibition of HIF1α blocked hypoxia-induced CCDC109B both in vitro and in vivo, indicating that HIF1α could regulate CCDC109B expression. Silencing of CCDC109B decreased hypoxia-induced migration and invasion. However, the underlying mechanisms in CCDC109B-mediated glioma invasion/migration under hypoxic conditions remains not fully clear. Further examination of regulation of HIF1α under normoxia and hypoxia may provide additional insight into its in GBM pathophysiology [37] and interacting factors may provide alternative therapeutic targets for the treatment of GBM.

## Conclusions

In summary, we discovered a potential role for CCDC109B as an oncogene and prognostic marker in human glioma. However, the mechanisms of CCDC109B in mediating glioma progression and possibly other human cancers remains to be investigated.

## Additional files

**Additional file 1: Table S1.** Association of *HIF1α* expression with *CCDC109B* expression in GBM patients.

**Additional file 2: Figure S1.** *HIF1α* and *CCDC109B* mRNAs levels decreased in cells treated with HIF1α siRNA. (A-B) U87MG and U251 cells were treated with NC, si-HIF1α-1 or si-HIF1α-2 under normoxia or hypoxia for 48 h. Expression levels of *HIF1α* and *CCDC109B* were determined using qRT-PCR. (**P < 0.01, ***P < 0.001).

## Abbreviations

MMP2: matrix metallopeptidase 2
MMP9: matrix metallopeptidase 9
