# Supplementary material for: Coiled-coil domain containing 109B is a HIF1α-regulated gene critical for progression of human gliomas
Source: J Transl Med. 2017 Jul 28;15:165. doi: 10.1186/s12967-017-1266-9 (PMC5534085; doi:10.1186/s12967-017-1266-9)
Supplement: Supplementary file 1 — Additional file 1: Table S1. Association of HIF1α expression with CCDC109B expression in GBM patients. [file 12967_2017_1266_MOESM1_ESM.docx]

|  | | HIF1α expression | | *P* value |
| --- | --- | --- | --- | --- |
|  |  | Weak | Strong |  |
| CCDC109B expression | Weak | 8 | 5 | 0.020 |
|  | Strong | 4 | 15 |  |

**Table S1. Association of *HIF1α* expression with *CCDC109B* expression in GBM patients (*n* = 32).**
