# Supplementary figures and images for: Coiled-coil domain containing 109B is a HIF1α-regulated gene critical for progression of human gliomas
Source: J Transl Med. 2017 Jul 28;15:165. doi: 10.1186/s12967-017-1266-9 (PMC5534085; doi:10.1186/s12967-017-1266-9)

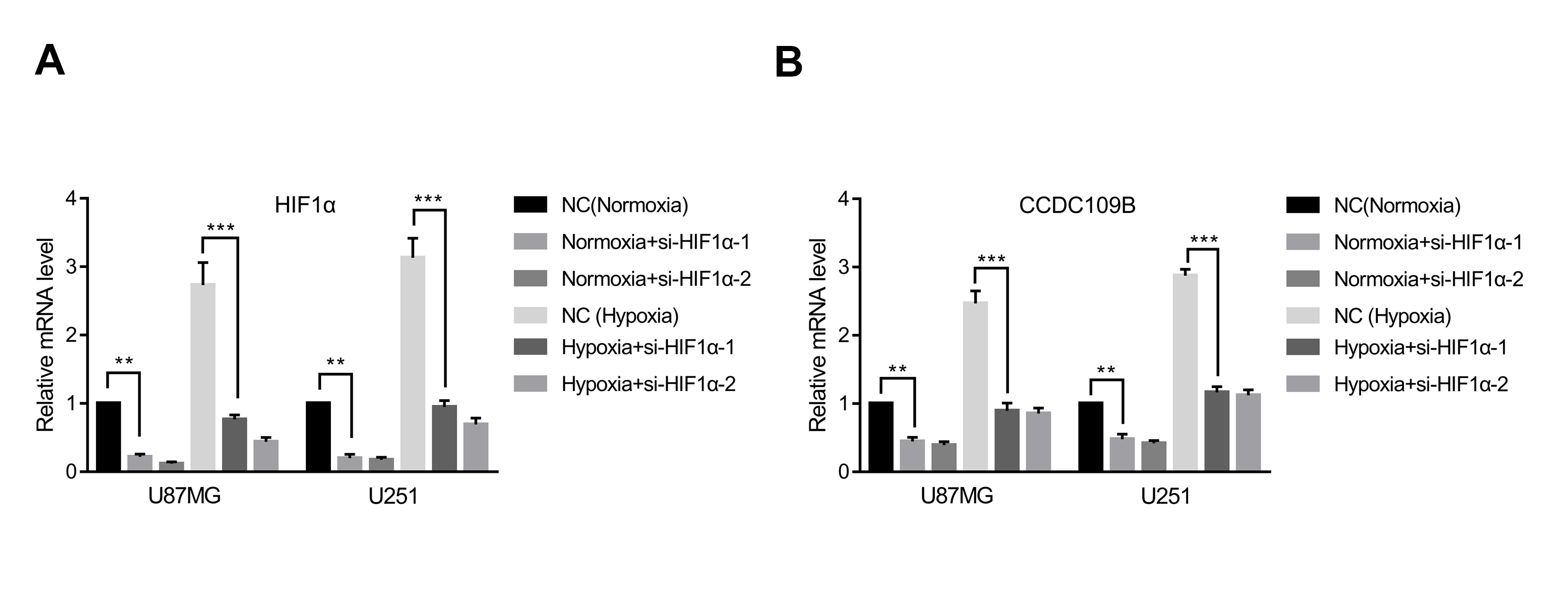

Supplement: Supplementary file 2 — Additional file 2: Figure S1. HIF1α and CCDC109B mRNAs levels decreased in cells treated with HIF1α siRNA. (A-B) U87MG and U251 cells were treated with NC, si-HIF1α-1 or si-HIF1α-2 under normoxia or hypoxia for 48 h. Expression levels of HIF1α and CCDC109B were determined using qRT-PCR. (**P < 0.01, ***P < 0.001). [file 12967_2017_1266_MOESM2_ESM.tif]
